# Safeguarding LGBTQ+ lives in an epoch of abandonment

**DOI:** 10.1016/S2214-109X(23)00353-4

**Published:** 2023-09

**Authors:** William E Rosa, Nicholas Metheny, Alic G Shook, Adebola A Adedimeji

**Affiliations:** Department of Psychiatry and Behavioral Sciences, Memorial Sloan Kettering Cancer Center, New York, NY, USA; School of Nursing and Health Studies, University of Miami, Coral Gables, FL, USA; College of Nursing, Seattle University, Seattle, WA, USA; Division of Health Behavior Research and Implementation Science, Department of Epidemiology and Population Health, Albert Einstein College of Medicine, New York, NY, USA; Diversity, Equity, and Inclusion Initiative, Montefiore Einstein Cancer Center, New York, NY, USA

Policy-sanctioned threats to the lives and welfare of LGBTQ+ people are becoming increasingly volatile. Despite the commitment of UN member states to “leave no one behind” as a key ethic of the Sustainable Development Agenda,^1^ LGBTQ+ populations are being actively marginalised further. 66 jurisdictions worldwide criminalise private, consensual, same-sex activity between men; 41 countries enact the same punishment for women; and 12 countries impose or can impose the death penalty for same-sex activity.^2^ Another 14 countries criminalise trans people’s gender identity or expression.^2^ An emerging global movement of homophobia, transphobia, and human-rights violations^3^ is pervading throughout countless nations, regardless of their income level, predominant religious practices, or governments.

On June 5, 2023, the Human Rights Campaign declared a state of emergency for all LGBTQ+ Americans, citing 525 state-level bills targeting LGBTQ+ populations in the USA.^4^ In 2023, 75 such bills have been signed into law—doubling legalised discrimination since 2022.^4^ These policies restrict educators from discussing LGBTQ+ issues, prohibit mental health workers from providing gender-affirming care to people under the age of 18 who have gender dysphoria, and criminalise parents who seek the medical care that they believe will best support the wellbeing their children. In Florida, the Protections of Medical Conscience Act will permit health workers to deny LGBTQ+ people health services of any kind if clinicians feel that these services violate their moral, ethical, or religious beliefs.^5^ This act creates a dangerous precedent of restricting health-care access and delivery based on the self-determined moral and ethical responsibility of individual clinicians.

Some countries are brashly expanding their longterm anti-LGBTQ+ legislation. For example, the Anti-Homosexuality Bill was signed into Ugandan law on May 29, 2023.^6^ The bill reiterates the existing punishment of lifelong imprisonment for same-sex conduct and decrees a new death penalty for “aggravated homosexuality”, which includes “serial offenders” and individuals engaged in same-sex activity with people who have disabilities.^6^ The bill also calls for a 20-year prison sentence for anyone “promoting homosexuality” (eg, anyone who advertises or distributes relevant material) and fines or imprisons people who fail to report a suspect having same-sex relations.^6^

Political instability increases the risk of harm for LGBTQ+ people. For example, since the Taliban took control of Afghanistan in 2021, they have systematically pursued and punished members of the LGBTQ+ community.^7^ Many victims, predominantly gay men and trans women, have faced Taliban-led physical and sexual assault, detention, and public humiliation (eg, stoning or flogging).^7^ In-country assistance and aid are commonly unavailable and escape for LGBTQ+ citizens is often impossible. Structural limitations on the freedom of movement for people seeking to flee identity-motivated violence (eg, economic restraints, an inability to obtain visas, or unclear asylum policies) lead to unspeakable suffering for thousands of LGBTQ+ people.^8^

LGBTQ+ marginalisation is evident even in end-of-life settings, in which people with serious illness or injury are most fragile. The end-of-life experiences of LGBTQ+ people are often characterised by fear of discrimination, heteronormative and cisnormative assumptions, homophobia and transphobia, social isolation, and undignified death.^9^ Too many trans people prefer suicide instead of the loss of functional independence as they are concerned that reliance on health workers and a lack of decision-making capacity will lead to mistreatment and violence.^9^ Spouses and partners of LGBTQ+ people who have died experience additional bereavement stressors (eg, disenfranchised grief or unwanted disclosure of identity),^10^ meaning they are at risk for prolonged grief disorder, which has further detrimental physical and mental health outcomes.

Affirmative practices to create more equitable health and social cultures for LGBTQ+ people focus on more inclusive health communication,^10^ consistent dismantling of heteronormativity and cisnormativity,^10^ accurate documentation of sexual orientation and gender identity (based on the safety of the clinical and social environment),^9,10^ and continued health advocacy to promote access to high-quality and affordable health services throughout the lifespan.^4^ Additional calls to action to further safeguard the lives and welfare of LGBTQ+ people are urgently needed (panel). Despite some hopeful policy wins (eg, decisions in Namibia and Taiwan to expand LGBTQ+ rights in 2023),^11,12^ much of the world has entered an epoch of LGBTQ+ population abandonment—a society in which the visibility of LGBTQ+ people is reduced, their health care is subpar, their welfare is dispensable, and their existence is undervalued. Unified commitments that reflect the core values of the Sustainable Development Goals^1^ and basic human rights^3^ have never been more crucial. The welfare of LGBTQ+ lives is everyone’s responsibility. We cannot, and will not, be left behind.

## Panel: Immediate calls to action to safeguard the lives and welfare of LGBTQ+ people

- UN member states should be held to account for enacting LGBTQ+ anti-discrimination policies that contradict their support of the Sustainable Development Goals
- Ministries of health and social welfare should advocate for the provision of people-centred services that are inclusive, protective, and responsive to the cultural needs and social and political risks of LGBTQ+ populations
- Violence that targets LGBTQ+ people should be legally prosecuted; perpetrators of violence should be accountable for inhumane and unjustified behaviour
- Health workers should abide by their ethical commitments of beneficence (ie, doing good), non-maleficence (ie, doing no harm), and justice regardless of the sexual orientation or gender identity of the patient
- Health systems and their employees should promote cultural, social, psychological, and physical safety for LGBTQ+ people and their families who seek medical care (eg, avoid heteronormative and cisnormative assumptions and language, ensure policies are inclusive and respectful, include chosen families in care planning and shared decision making, and affirm allyship and safety by displaying LGBTQ+ pride symbols)
- Senior health leaders and administrators should consistently and empathically acknowledge, validate, and normalise LGBTQ+ people’s lived experiences both publicly and privately, and should guide other employees and trainees accordingly
- Non-governmental organisations and community-based groups that advocate for the rights of LGBTQ+ people should be given social and political protections to sustain activities and expand outreach to LGBTQ+ citizens, families, and allies
- Researchers should partner with LGBTQ+ communities and advocates to prioritise research aims that will directly benefit community needs and priorities (eg, community-based participatory approaches)
- Faith leaders and religious communities should consider their potential contributions to perpetuating stigma and should redesign their influence to aim toward a nonviolent and inclusive civil society
- Businesses and employers should consistently be examples of allyship by not tolerating discriminatory practices and partnering with LGBTQ+ people as coworkers and colleagues
- Citizens, neighbours, and family members of LGBTQ+ people should continue to participate in advocacy, activism, and allyship that shows solidarity and support for inclusive and humane communities
- *Lancet* Commission agendas and commissioners should promote respectful discourse on LGBTQ+ inclusion and visibility when addressing the needs of marginalised groups and finalising report implications for health policy and practice
